# Shen-ling-bai-zhu-san ameliorates inflammation and lung injury by increasing the gut microbiota in the murine model of Streptococcus pneumonia-induced pneumonia

**DOI:** 10.1186/s12906-020-02958-9

**Published:** 2020-05-27

**Authors:** Jinli Feng, Weibo Dai, Cheng Zhang, Houjun Chen, Ziliang Chen, Yongfeng Chen, Qianyi Pan, Yongmao Zhou

**Affiliations:** 1 Emergency department, Zhongshan Hospital of traditional Chinese Medicine, No. 3 Kangxin road, west district, Zhongshan, Guangdong 528401 People’s Republic of China; 2 Pharmacology laboratory, Zhongshan Hospital of traditional Chinese Medicine, Zhongshan, Guangdong 528401 People’s Republic of China; 3 Clinical laboratory, Zhongshan Hospital of traditional Chinese Medicine, Zhongshan, Guangdong 528401 People’s Republic of China; 4 Prevention and health section, Zhongshan Hospital of traditional Chinese Medicine, Zhongshan, Guangdong 528401 People’s Republic of China; 5 Pediatrics, Zhongshan Hospital of traditional Chinese Medicine, Zhongshan, Guangdong 528401 People’s Republic of China

**Keywords:** Gut microbiota, Inflammation, Lung injury, Shen-ling-bai-zhu-san, *Streptococcus pneumoniae*

## Abstract

**Background:**

Shen-ling-bai-zhu-san (SLBZS) regulates inflammation and gut microbiota which are associated with *Streptococcus pneumoniae* (*Spn*)-induced pneumonia. So, we studied the therapeutic effect of SLBZS and evaluated whether gut microbiota is associated with the effects of SLBZS in improving *Spn*-induced pneumonia.

**Methods:**

*Spn*-induced pneumonia NIH mice were treated by SLBZS and cefixime. A CT scan was performed and Myeloperoxidase (MPO) activity in lung homogenates was determined using the MPO Colorimetric Assay Kit. Inflammation levels in lung homogenates were measured using ELISA. Bacterial load was coated on a TSAII sheep blood agar. Intestinal gut microbiota information was analyzed according to sequencing libraries.

**Results:**

SLBZS decreased bacterial load, reduced wet/dry weight ratio, inhibited myeloperoxidase activity, reduced the neutrophils count, and ameliorated lung injury. Furthermore, SLBZS inhibited interleukin (IL)-1β, IL-6, tumor necrosis factor-α, IL-2, IL-8, IL-12, and interferon-γ secretion and enhanced IL-10 secretion. These results suggest that SLBZS ameliorates lung injury in mice with *Spn*-induced pneumonia. Moreover, SLBZS reduced inflammatory cytokine levels in a concentration-dependent manner and increased gut microbiota abundance and diversity. After SLBZS treatment, bacteria such as Epsilonbacteraeota, Bacteroidetes, Actinobacteria, Proteobacteria, and Patescibacteria were significantly reduced, while Tenericutes and Firmicutes were significantly increased.

**Conclusion:**

SLBZS ameliorates inflammation, lung injury, and gut microbiota in mice with *S. pneumoniae*-induced pneumonia.

## Background

Pneumonia, which is an inflammatory disease caused by bacteria, viruses, fungi, or other microorganisms, mainly affects children and the elderly and has high morbidity and mortality worldwide, and the morbidity and mortality of pneumonia is increasing [1–3]. *Streptococcus pneumoniae* (*Spn*), a gram-positive bacterium with over 90 serotypes, is the most common bacterial pneumonia-causing pathogen [4]. Ever since the introduction of pneumococcal vaccine, morbidity and mortality of pneumonia have reduced significantly; however, global coverage of vaccine is inadequate [5]. Current pneumonia therapeutic strategies, including antimicrobial regimens, antibiotics, and adjunctive therapies, often lead to drug resistance [6]. Thus, it is necessary to find alternative drugs for the alleviation of pneumonia-induced tissue injury and death.

Traditional Chinese medicine (TCM) therapy which have the unique profile of multiple target can decrease risks associated with pneumonia. A previous study revealed that Buzhong Yiqi decoction and XueBiJing Injection could reduce the incidence of pneumonia in patients with Severe Community-Acquired Pneumonia [7, 8]. TCM therapy alone or in combination with antibiotics reduces treatment failure, time to clinical stability, length of hospital stay, and in-hospital mortality and improves quality of life in community acquired pneumonia patients [9, 10]. TCM may become a promising new option for pneumonia. Shen-ling-bai-zhu-san (SLBZS) is a TCM which has been extensively used in China. SLBZS improves hypoglycemic action and β-cell function, offers protection against ulcerative colitis, and alleviates inflammation and non-alcoholic steatohepatitis-induced liver injury, [11–13]. Additionally, SLBZS can promote beneficial gut microbiota abundance [14]. Previous study found that the distribution of intestinal flora is closely related to pneumonia. Neonatal gut microbiota colonization can regulate lung immunity to prevent pneumonia, plays an important role in the “gut-lung axis” [15]. In pneumonia patients, changes in gut microbiota such as *Bifidobacterium* and *Escherichia coli* have been reported [16]. Gut microbiota enhances primary alveolar macrophage function, enabling them to act as a protective mediator during pneumococcal pneumonia infection [17]. A clinic trial showed that Oral probiotic preparation containing Lactobacillus, Bifidobacterium, and Streptococcus spp. decreased the length of ICU and hospital stay and the incidence of ventilator-associated pneumonia [18]. However, it remains unclear whether SLBZS can improve lung injury and treat *Spn*-induced pneumonia via regulating gut microbiota.

Therefore, the aim of this study was to investigate the therapeutic effect of SLBZS on *Spn*-induced pneumonia National Institutes of Health (NIH) mice, as well as whether gut microbiota is associated with the effects of SLBZS in *Spn*-induced pneumonia improvement. Furthermore, cefixime is used as a positive control. Cefixime is a third-generation cephalosporin antibiotics which plays bactericidal effect. Cefixime is used to treat pneumonia because of it has broad spectrum activity to put down all Gram-negative and positive pathogens and atypical organisms, e.g. Mycoplasma and Chlamydia [19]. By comparing with the treatment effect of cefixime, the effect and advantages of SLBZS in treating pneumonia were clarified.

## Methods

### Preparation of the decoction of SLBZS

SLBZS was purchased from Tongrentang (SFDA approval number: Z1102O755; Beijing, China) which includes: Lian zi (Nelumbinis Semen), Yi Ren (Coicis Semen), Sha ren (Amomi Fructus), Jie geng (Platycodonis Radix), Bai bian dou (Lablab Semen Album), Fu Ling (Poria), Gan cao (Glycyrrhizae Radixet Rhizoma), Bai Zhu (Macrocephalae Rhizoma), Shan Yao (Dioscoreae Rhizoma), Dang shen (*Codonopsis pilosula*), and Dazao (Fructus Ziziphi Jujubae), and the ratio was 5:5:5:5:7.5:10:10:10:10:10:5. It was dissolved in distilled water before use in different experiments. Additionally, the usual dosage of SLBZS in adults is 6 ~ 9 g/day (Adult weight: 70KG). and according to “The algorithm of drug dosage exchange between different kinds of animals” (the algorithm of drug dosage exchange between human and mouse is 9.1), the low dosage of SLBZS (low-SLBZS) in mice was 0.86 g/kg. Considering the multiplication factors of 2 and 4, the medium dose (medium-SLBZS) and high dose (high-SLBZS) were determined to be 1.72 and 3.44 g/kg, respectively, according to low dosage of SLBZS (0.86 g/kg).

### Mice feeding

All animal protocols were approved by the ethics committee of the Zhongshan Hospital, Guangzhou University of Chinese Medicine (approved No.: 201804; Zhongshan, China), and disposal methods were in accordance with animal ethics standards. Sixty male specific pathogen-free NIH mice, weighing 13–15 g were purchased from Guangdong Medical Laboratory Animal Center. They were then housed in a continuously ventilated room under a 12 h/12 h light/dark cycle at a controlled temperature (between 20 and 25 °C), with free access to water and food. The mice were allowed to adapt to the environment for 7 days before the establishment of the *Spn*-induced pneumonia mice model.

### *Spn*-induced pneumonia mice

*Spn*-induced pneumonia mice models were established as previously described [20]. *Spn* (ATCC49619, American Type Culture Collection, Rockville, MD, USA) was inoculated overnight on TSAII sheep blood agar (Nihon Becton Dickinson, Tokyo, Japan), and cultured for 18 h at 37 °C in 5% CO_2_. The bacteria were then harvested by centrifugation and re-suspended in sterile phosphate-buffered saline (0.15 M, pH 7.2, PBS) to 10^9^ CFU (colony-forming unit)/ml. Mice were briefly anesthetized by inhalation of 3% isoflurane. Thereafter, 100 μl PBS containing 1 × 10^8^ CFU was inoculated into both nostrils of each mice using a 29-gauge needle, so as to establish mice models. Mice in the normal group, for prophylactic protocol, were administered PBS, without infection.

### Experimental design and drug administration

**Fig. 1:**
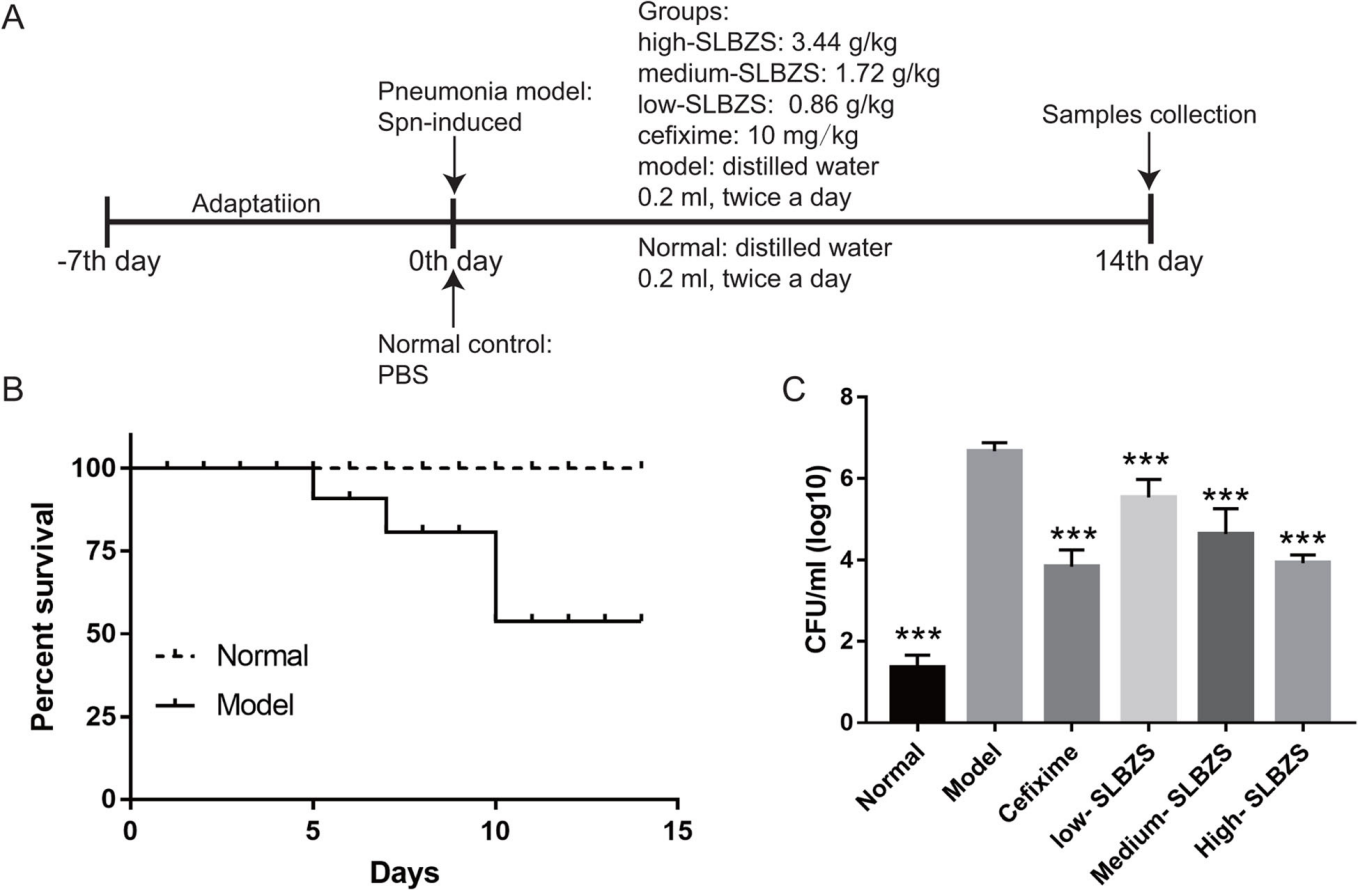
SLBZS treatment improves survival and ameliorates bacterial load in the broncho-alveolar lavage fluid of mice with *Spn*-induced pneumonia. **a** flow chart; **b** survival rate in normal and model group. Note: Survival rate in four treatment group same with survival rate in normal group, so the survival rate in four treatment group were not shown. **c** Bacterial load in the broncho-alveolar lavage fluid. ***p < 0.05 vs. the model group. CFUs, colony-forming units. Number mice in normal, model, high-SLBZS, medium-SLBZS, low-SLBZS, cefixime granules groups were 10, 6, 10, 10, 10, and 10, respectively. Each experiment was repeat three times

As shown on the flow chart of the experiment (Fig. 1a), the *Spn*-induced pneumonia mice (*n* = 50) were randomly divided into 5 groups (*n* = 10) as follows: high-SLBZS, medium-SLBZS, low-SLBZS, cefixime granules (cefixime group, Product ID: B14202031187; SFDA approval number: H10950235; Baiyunshan pharmaceutical general factory, Guangzhuo, China), and the model group. For treatment, cefixime granules was prepared to a 1 mg/ml solution and the treatment concentration was 10 mg/kg. The mice in each group were intragastrically administered a 0.2 ml dose of the corresponding test substance twice a day for 14 days. For the normal (*n* = 10) and model groups, the test substances were replaced by an equal volume of distilled water. After the 14-day treatment, feces samples were collected (from 8:00 am to 16:00 pm) using metabolic cages with ice-packed Eppendorf tubes, and immediately stored at –80 °C until analysis. At the end of the treatment, all the mice were anesthetized after an overnight fast, and serum samples were collected from the orbital plexus, while lung tissues were harvested for further analysis. After treatment at 14 days, *Spn*-induced pneumonia mice were sacrificed by an intraperitoneal injection of 3% sodium pentobarbital (120 mg/kg of animal body weight).

### Lung injury and inflammation evaluation

A CT scan was performed using a GE LightSpeed VCT (GE Healthcare, Little Chalfont, UK), as previously described [21]. Mouse lungs were homogenized in Hank’s buffered salt solution, the slurry was centrifuged, and the supernatant was collected. Myeloperoxidase (MPO) activity in lung homogenates was determined using the MPO Colorimetric Assay Kit (Elabscience, Hangzhou, China). interferon (IFN)-γ, tumor necrosis factor (TNF)-α, interleukin (IL)-1β, IL-2, IL-12, IL-8, IL-6, and IL-10 levels in lung homogenates were measured using Enzyme-Linked Immunosorbent Assay (ELISA). Mouse IL-1β ELISA Kit was purchased from Elabscience (NO.: E-EL-M0037c, Hangzhou, China), and IFN-γ (No.: bsk12001), TNF-α (NO.: bsk12002), IL-2 (No.: bsk12016), IL-12 (No.: bsk12020), IL-8 (NO.: bsk12005), IL-6 (NO.: bsk12004), and IL-10 (NO.: bsk12007) ELISA Kits were purchased from Bioss (Beijing, China). Wet/dry weight ratio was calculated by intratracheal lavage, as previously describe d[21]. After treatment, the lung lobes of each mouse were weighed (wet weight), placed in an oven, and weighed daily until the weight remained unchanged (dry weight). The wet/dry weight ratios were calculated from the initial and final values. Hematoxylin and eosin (H&E) staining was performed as previously describe d[21]. Briefly, the diaphragmatic leaves of the right lung of mice from each group were fixed with 4% formalin, washed, dehydrated, embedded in paraffin. Then cut into 4-μm sections. H&E staining was performed using a kit (Solarbio, Beijing, China), and lung tissue morphology was observed under an optical microscope (200×, Olympus, Japan).

### Measure of bacterial load

After the 14-day treatment, mice were anesthetized with 3% pentobarbital sodium (P3761; Sigma-Aldrich, St Louis, MO, USA), and the broncho-alveolar lavage fluid (BALF) of the mice in each group was obtained by intratracheal lavage, as previously described [22]. The collected BALF (0.8 mL) was then centrifuged at 4 °C for 10 min at 260×*g*, and the precipitate re-suspended in 0.5 mL sterile PBS. Subsequently, the suspension was diluted in serial 10-fold dilutions, and the final diluent (50 mL) was coated on a TSAII sheep blood agar (Nihon Becton Dickinson), and fixed in 5% CO_2_ at 37 °C. Colony-forming units were counted 18 h later. The total protein content of BALF using a BCA protein assay (Beyotime, Beijing, China). And eosinophils, basophil, and neutrophils were counted by Giemsa stain (Leagene Biotechnology, Anhui, China).

### Intestinal gut microbiota information analysis

Intestinal gut microbiota information analysis was performed as previously described [23, 24]. Briefly, stool DNA was extracted from the precipitates using the E.Z.N.A.® Stool DNA Kit (Omega, Norcross, GA, USA). Thereafter, 16S rRNA Bac 16S genes: V3-V4 were amplified using 338F and 806R primers. PCR products were detected by 1% agarose gel electrophoresis and then mixed in equidensity ratios, according to the GeneTools Analysis Software v4.03.05.0 (SynGene). The mixture of PCR products was then purified using the E.Z.N.A. Gel Extraction Kit (Omega, Omega, Norcross, GA, USA). Sequencing libraries were generated using the NEBNext® Ultra™ DNA Library Prep Kit for Illumina® (New England Biolabs, MA, USA), and index codes were added. The library quality was assessed using the Qubit@ 2.0 Fluorometer (Thermo Fisher Scientific, MA, USA) and the Agilent Bioanalyzer 2100 system (Agilent Technologies, Waldbron, Germany). Finally, the library was sequenced on an IlluminaHiseq 2500 platform, and 250 bp paired-end reads were generated (Guangdong Magigene Biotechnology Co., Ltd. Guangzhou, China). Intestinal gut microbiota information was analyzed according to sequencing libraries.

### Statistical analysis

Statistical analysis was performed using the Statistical Package for the Social Sciences, version 19.0 (IBM, Armonk, NY, USA). Normally distributed data were expressed as the mean ± standard deviation Multiple groups were compared using one-way analysis of variance (ANOVA), followed by post-hoc tests of the Tukey. *P* values of < 0.05 were considered statistically significant.

## Results

### SLBZS ameliorates bacterial load and improves survival in *Spn*-induced pneumonia mice

The survival rates showed that 4 mice in the model group died on days 5 (1 mice), 7 (1 mice), and 10 (2 mice) after *Spn*-induced pneumonia. SLBZS and cefixime treatment improved survival in *Spn*-induced pneumonia mice (Fig. 1b). So, number mice in control, model, high-SLBZS, medium-SLBZS, low-SLBZS, cefixime granules groups were 10, 6, 10, 10, 10, and 10, respectively. Additionally, the bacterial load in BALF was significantly reduced in treatment groups, compared to that of the model group (Fig. 1c).

### SLBZS ameliorates lung injury and MPO activity in *Spn*-induced pneumonia mice

**Fig. 2:**
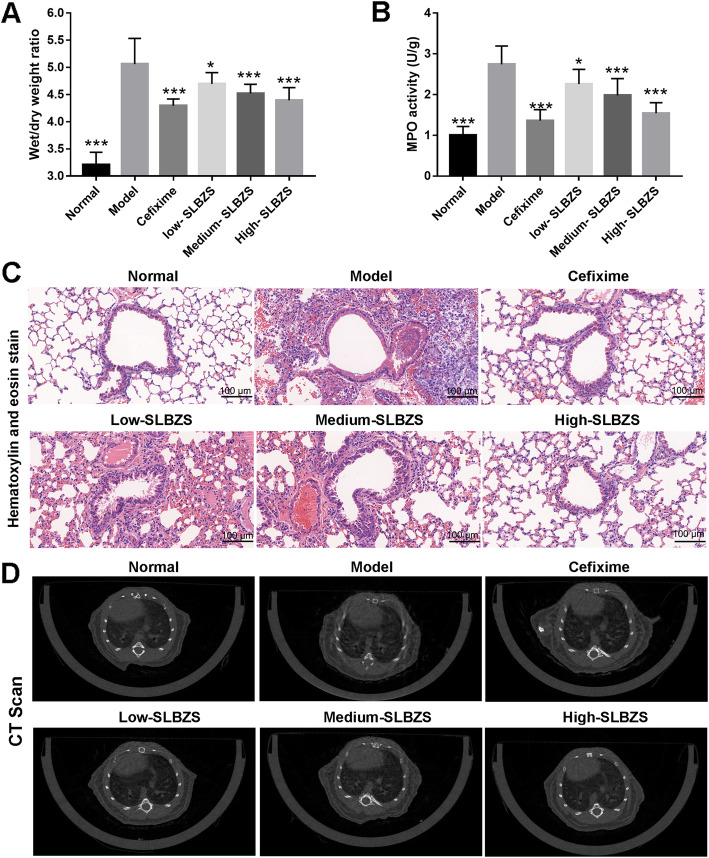
SLBZS treatment ameliorates lung injury in mice with *Spn-*induced pneumonia. **a** Wet/dry weight ratio was detected in *Spn-*induced pneumonia mice after treatment at 14 days. **b** MPO activity measured in *Spn-*induced pneumonia mice after treatment at 14 days. **c** Histomorphological analysis of lung tissue injury in *Spn-*induced pneumonia mice using hematoxylin and eosin staining after treatment at 14 days (magnification × 200). **d** Lung injury in *Spn-*induced pneumonia mice measured using CT scanning after treatment at 14 days. *p < 0.05 and ***p < 0.001 vs. the model group. Number mice in normal, model, high-SLBZS, medium-SLBZS, low-SLBZS, cefixime granules groups were 10, 6, 10, 10, 10, and 10, respectively. Each experiment was repeat three times

**Fig. 3:**
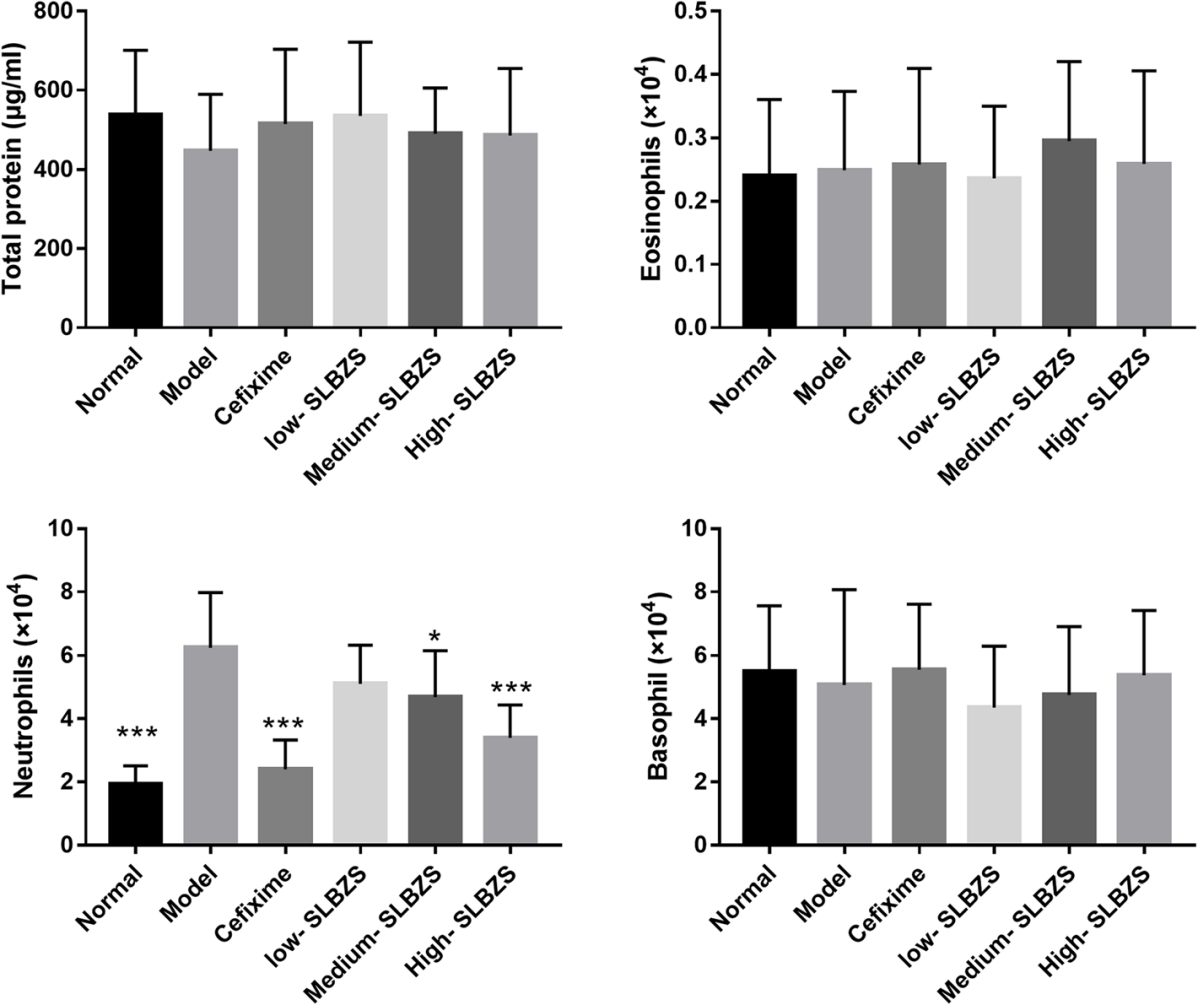
SLBZS treatment reduced the neutrophils count while had no significantly effect on total protein, eosinophils, and basophil in BALF. *p < 0.05, ***p < 0.001 vs. the model group. Number mice in normal, model, high-SLBZS, medium-SLBZS, low-SLBZS, cefixime granules groups were 10, 6, 10, 10, 10, and 10, respectively. Each experiment was repeat three times

Based on wet/dry weight ratio findings (Fig. 2a), the wet/dry weight ratio of model group mice increased significantly compared with that of the normal group, while those of mice in the 4 treatment groups were significantly lower than those of mice in the model group. In the model group, MPO activity was increased significantly, while in the SLBZS-treated groups, it was significantly reduced after treatment at 14 days (Fig. 2b). H&E staining and CT scanning results revealed the presence of inflammatory cell infiltration, alveolar collapse, and perivascular and peribronchial edema in the lung tissues of *Spn*-induced pneumonia mice. However, these states were reversed after cefixime and SLBZS treatment on day-14 (Fig. 2c and d). Additionally, cefixime and medium- and high-SLBZS treatment significantly reduced the neutrophils count while had no significantly effect on total protein, eosinophils, and basophil in BALF (Fig. 3). These results suggest that SLBZS treatment reduced lung injury, MPO activity, and neutrophils count in a concentration-dependent manner.

### SLBZS decreases inflammatory cytokine levels in *Spn*-induced pneumonia mice

**Fig. 4:**
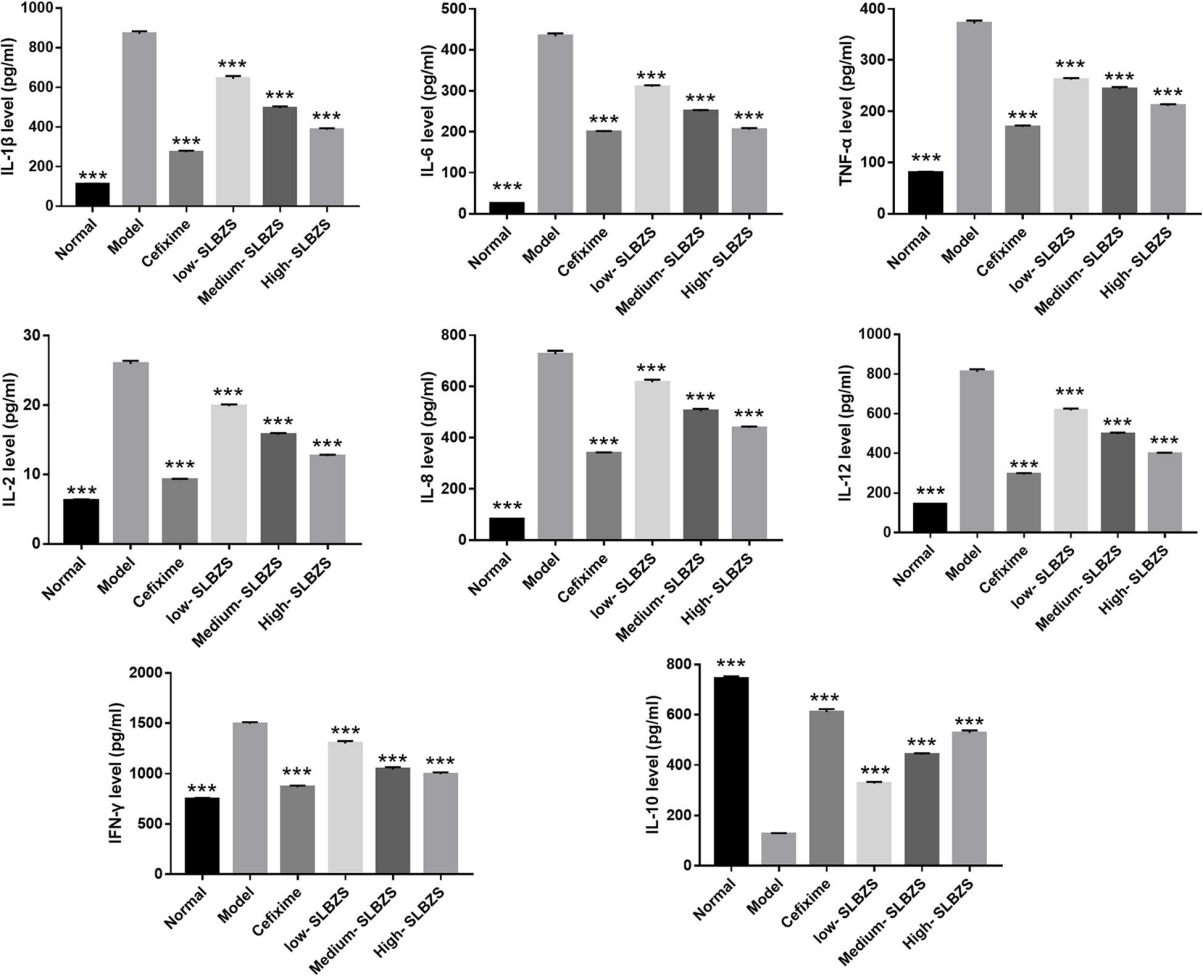
SLBZS treatment lowers inflammatory cytokine levels in *Spn*-induced pneumonia mice. IFN-γ, TNF-α, IL-1β, IL-2, IL-12, IL-8, IL-6, and IL-10 levels in lung homogenates measured using Enzyme-Linked Immunosorbent Assay. ***p < 0.001 vs. the model group. Number mice in normal, model, high-SLBZS, medium-SLBZS, low-SLBZS, cefixime granules groups were 10, 6, 10, 10, 10, and 10, respectively. Each experiment was repeat three times

Measurement of inflammatory cytokine levels using ELISA showed that IL-1β, IL-6, TNF-α, IL-2, IL-8, IL-12, and IFN-γ levels were significantly enhanced, while IL-10 levels were significantly reduced in the lung homogenates of *Spn*-induced pneumonia mice, compared with the normal mice (Fig. 4). Further, IL-1β, IL-6, TNF-α, IL-2, IL-8, IL-12, and IFN-γ levels were significantly reduced, while IL-10 levels were significantly enhanced in the lung homogenates of mice in the 4 treatment groups, compared with the model group. Furthermore, SLBZS treatment reduced inflammatory cytokine levels in a concentration-dependent manner.

### SLBZS ameliorates gut microbiota in *Spn*-induced pneumonia mice

#### Operational taxonomic units

**Fig. 5:**
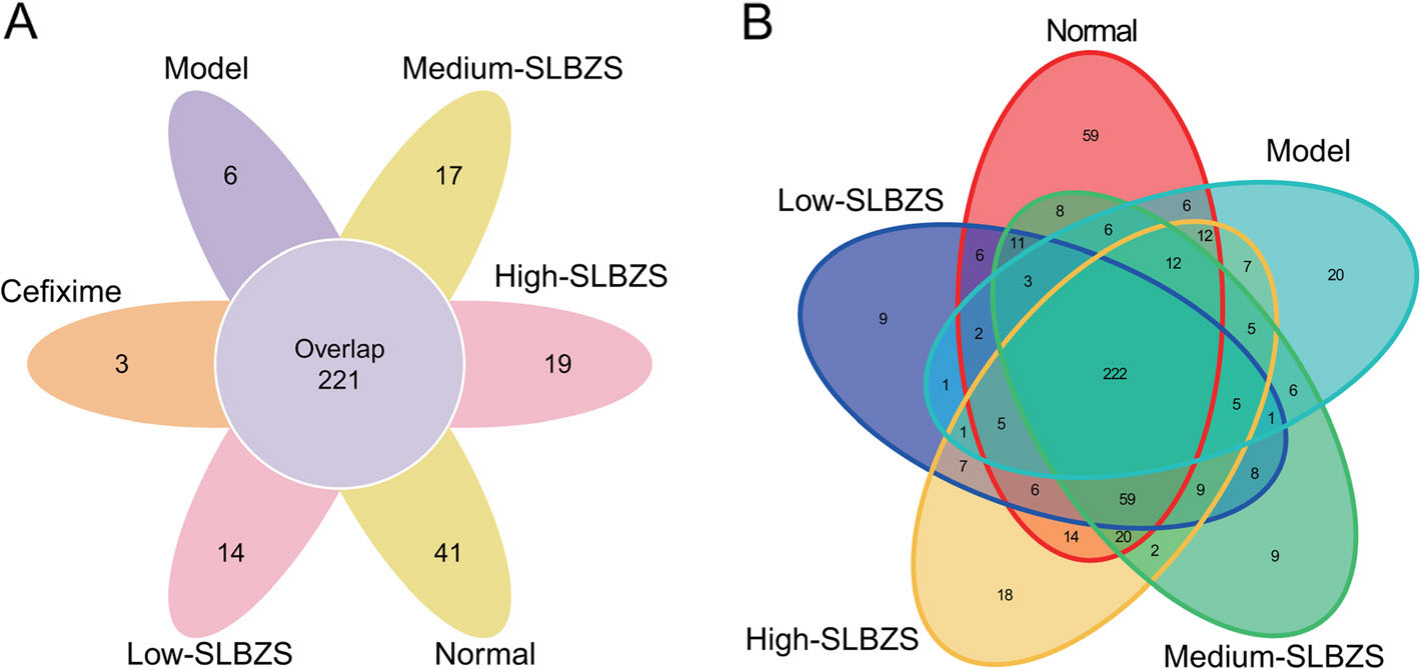
Operational Taxonomic Unit (OTU) distribution in each group was descripted using a Venn map. Different colors represent different samples. If for instance, 221 was simultaneously marked in 2 different circles, it meant that the two samples had 221 same sequences categorized in the same OTUs. **a** OTUs distribution in normal, model, cefixime, low-, medium-, and high-SLBZS groups was descripted using a Venn map. **b** OTUs distribution in normal, model, low-, medium-, and high-SLBZS groups was descripted using a Venn map. Number mice in normal, model, high-SLBZS, medium-SLBZS, low-SLBZS, cefixime granules groups were 3. Each experiment was repeat three times

The operational taxonomic units (OTUs) of intestinal in normal, model, cefixime, low-, medium-, and high-SLBZS groups were 451, 307, 257, 355, 386, and 406, respectively. The OTUs of intestinal in each group, as well as the overlapping OTUs in the different groups, were illustrated using a Venn picture. The results showed that 221 OTUs of intestinal contained normal, model, cefixime, low-, medium-, and high-SLBZS groups, while the specific OTUs in the 6 groups were 41, 6, 3, 14, 17, and 19, respectively (Fig. 5a). Additionally, 222 OTUs of intestinal contained normal, model, low-, medium-, and high-SLBZS groups, while the specific OTUs in the 5 groups were 59, 20, 9, 9, and 18, respectively (Fig. 5b).

#### Gut bacterial community diversity

**Fig. 6:**
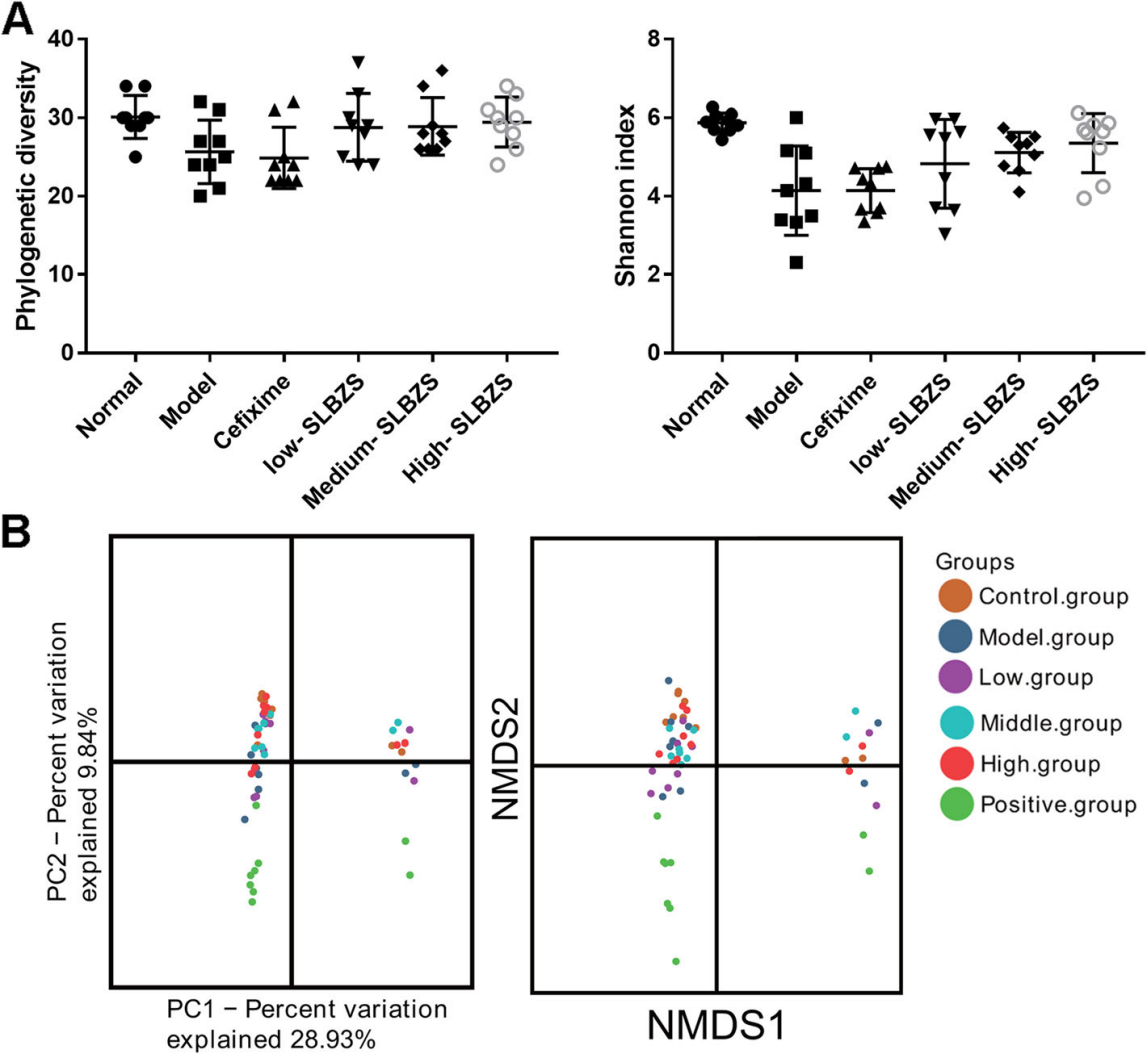
Alpha diversity. **a** Phylogenetic diversity and Shannon diversity of each group after treatment at 14 days. **b** Principal co-ordinates analysis (PCoA) and nonmetric multidimensional scaling (NMDS) reflected sample differences and distances on two-dimensional coordinate graphs. Points of different colors represent different groups, and the closer the sample spatial distance, the more similar the species composition of the sample is. Number mice in normal, model, high-SLBZS, medium-SLBZS, low-SLBZS, cefixime granules groups were 3. Each experiment was repeat three times

**Fig. 7:**
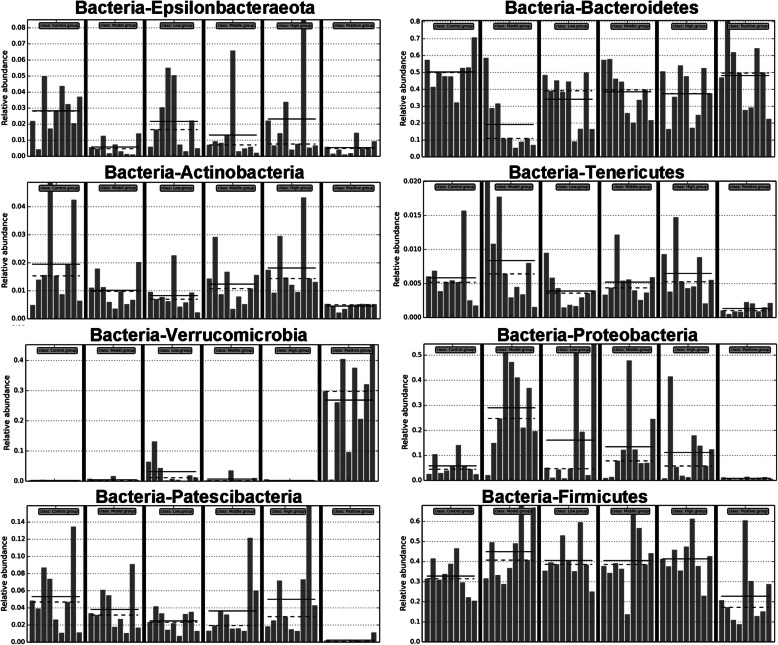
The bacteria species Epsilonbacteraeota, Bacteroidetes, Actinobacteria, Tenericutes, Verrucomicrobia, Proteobacteria, Patescibacteria, and Firmicutes were analyzed in all groups after 14 days treatment. Number mice in normal, model, high-SLBZS, medium-SLBZS, low-SLBZS, cefixime granules groups were 3. Each experiment was repeat three timess

Phylogenetic diversity and Shannon index were estimated to determine the microbiota ecological diversity of each sample (Fig. 6a). As expected, compared with the normal group, *Spn*-induced pneumonia mice had lower gut bacterial community diversity (lower phylogenetic diversity and Shannon index), which was enhanced by SLBZS treatment. However, compared with *Spn*-induced pneumonia mice, cefixime treatment did not significantly change gut bacterial diversity. Principal co-ordinates analysis reflected sample differences and distances on a two-dimensional coordinate graph by analyzing the differences of principal component 1 (PC1) and principal component 2 (PC2). Nonmetric multidimensional scaling (NMDS) analysis reflected sample differences and distances on a two-dimensional coordinate graph by analyzing the value differences based on evolution or number distance matrix. The results showed that the differences and distances between normal and high-SLBZS groups on the two-dimensional coordinate graph were significantly lower than those between the normal group and other groups (Fig. 6b). Additionally, gut bacterial community structure differences between the normal, model, and high-SLBZS groups were analyzed using the Anosim and Adonis analysis (Table 1), and the results showed that the gut bacterial community structure of the normal group was different from that of the model group; however, this difference could be reduced by high-SLBZS treatment. Further, gut bacterial species structure differences between the normal, model, and high-SLBZS groups were analyzed using the LDA Effect Size analysis (Fig. 7). Compared with the normal group, the gut bacteria species Epsilonbacteraeota, Bacteroidetes, Actinobacteria, Proteobacteria, and Patescibacteria were significantly reduced; Tenericutes and Firmicutes were significantly increased; and Verrucomicrobia was not significantly changed in the model group. These gut microbiota changes were reversed by SLBZS treatment.

**Table 1:** The community structure differences between normal, model, and high-SLBZS groups analyzed using Anosim and Adonis analysis

Group		Analysis Value	P
Anosim analysis	Normal: Model	R = 0.618	0.001
	Model: High-SLBZS	R = 0.327	0.008
	Normal: High-SLBZS	R = 0.135	0.059
Adonis analysis	Normal: Model	R^2^ = 0.329	0.001
	Model: High-SLBZS	R^2^ = 0197	0.01
	Normal: High-SLBZS	R^2^ = 0.116	0.024

## Discussion

Current *Spn*-induced pneumonia antibiotic therapies often lead to drug resistance. Thus, it is important to find alternative drugs for the alleviation of pneumonia-induced deaths. SLBZS is a well-known TCM formula used in China for the treatment of gastrointestinal disorders such as diarrhea, ulcerative colitis, and inflammatory bowel disease (IBD) [13, 25, 26]. This study showed that SLBZS or cefixime treatment decreased bacterial load in BALF, reduced wet/dry weight ratio, inhibited MPO activity and reduced neutrophils count in BALF, and ameliorated lung injury in *Spn*-induced pneumonia, suggesting that these treatments ameliorates lung injury in *Spn*-induced pneumonia mice. Additionally, we found that high dosage SLBZS treatment and cefixime treatment have similar treatment effects, which provides new insights regarding the use of TCM in *Spn*-induced pneumonia treatment. Microbial balance in the specific tissue was associated with the development of disease [27]. Increasing studies have demonstrated that microbial diversity in the BLAF was significantly decreased and relationship with the disease severity in patients with pneumonia such as bacterial pneumonia, *Mycoplasma pneumoniae* pneumonia [28, 29]. In the BLAF of patients with *Spn*-induced pneumonia, *Spn* were accumulated and had low microbial diversity because *Spn* secreted the abundant various virulence factors to inhibit the other bacterial survival [30]. Additionally, good outcome had correlation with reduced bacterial load in the BLAF of *Spn*-induced pneumonia patients [31]. In this study, we found that SLBZS treatment decreased bacterial load in BALF of *Spn*-induced pneumonia patients, which may be a reason in improving lung injury. However, the effects of SLBZS treatment on microbial diversity in the BLAF of patients with *Spn*-induced pneumonia needs further study.

*Spn* infection promotes the secretion of inflammation cytokines, enhancing bacterial dissemination and disease severity. The key surface components of *Spn* include pneumolysin, peptidoglycan, teichoic acid, and hydrogen peroxide, which promoted inflammatory cytokine in lung tissue. Additionally, bacterial cell wall components promoted inflammation via the NF-κB signaling pathway, the activation of Nod receptors, and the release of C3a and C5a [32]. Levels of pro-inflammatory cytokines, including IL-1β, TNF-α, IL-6, IL-2, IL-8, IL-12, and IFN-γ, are increased during *Spn* infection; thus, enhancing lung injury [33]. IL-10 acts as an anti-inflammatory cytokine in bacterial infections [34]. A previous study showed that it inhibits neutrophil recruitment and lung inflammation, improving host survival during *Spn* infection, although it cannot efficiently prevent bacterial dissemination [35]. Its inhibition of inflammation contributes to respiratory barrier integrity restoration after *Spn* infection; hence, it could be used to design novel therapeutic interventions for *Spn* bacterial infection prevention. SLBZS treatment inhibited the TLR4/p38 MAPK signaling pathway, attenuating lipid metabolic disturbance, reducing IL-1β, TNF-α, and IL-6 levels, and reversing non-alcoholic steatohepatitis progression [12, 36]. In IBD, IL-1β and TNF-α levels are decreased, whereas that of IL-10 is increased after SLBZS treatment at day 7, thus hindering the development of IBD [26]. Similar to these findings, results of the present study revealed that SLBZS treatment inhibited IL-1β, IL-6, TNF-α, IL-2, IL-8, IL-12, and IFN-γ secretion, and promoted IL-10 secretion, suggesting that SLBZS treatment ameliorates lung injury in *Spn*-induced pneumonia mice by regulating inflammatory cytokine secretion. Moreover, SLBZS treatment reduced inflammatory cytokine levels in a concentration-dependent manner.

Current *Spn*-induced pneumonia antibiotic therapies often lead to drug resistance, which may be associated with host gut microbiota disruption [37, 38]. Gut microbiota, especially the presence of *segmented filamentous bacteria*, increased pulmonary type 17 immunity and reversed the methicillin-resistant of *Spn* [39, 40]. OTUs represent the gut microbiota abundance. The present study revealed that the OTUs of intestinal in normal, model, and cefixime groups were 451, 307, 257, respectively, suggest that cefixime treatment decrease the gut microbiota abundance, a finding that agrees with the results of previous studies [41]. SLBZS treatment can improve antibiotic-associated diarrhea by inducing gut microbiome structural changes, particularly Bacteroides spp. changes [42]. In IBD rats, SLBZS treatment reportedly induced the restoration of Corynebacteriaceae, Lactobacillaceae, Paraprevotellaceae, Veillonellaceae, Prevotellaceae*,* and Clostridiaceae levels to normal [26]. The OTUs of intestinal in normal, model, low-, medium-, and high-SLBZS groups were 451, 307, 355, 386, and 406, respectively, suggest that SLBZS treatment increased the gut microbiota abundance. Phylogenetic diversity and Shannon index were estimated to determine the microbiota ecological diversity of each sample. Gut microbiome diversity rise with increasing phylogenetic diversity and shannon index. The present study also revealed that SLBZS treatment increased phylogenetic diversity and shannon index, suggested that gut microbiome diversity was improved after SLBZS treatment in *Spn*-induced pneumonia mice. PCoA and NMDS reflected the species composition. The closer the sample spatial distance, the more similar the species composition of the sample is. Anosim and Adonis analysis reflected gut bacterial community structure. The similar of gut bacterial community structure increase with decreasing the value of Anosim and Adonis analysis. In this study, we found that SLBZS treatment decrease the sample spatial distance between high-SLBZS treatment group and normal group and reduced the value of Anosim and Adonis analysis. These results suggested that the species composition and gut bacterial community structure had high similarity between high-SLBZS treatment group and normal group. Additionally, the gut bacteria species Epsilonbacteraeota, Bacteroidetes, Actinobacteria, Proteobacteria, and Patescibacteria were significantly reduced, while Tenericutes and Firmicutes were significantly increased, after SLBZS treatment, suggesting that SLBZS treatment increases the gut microbiota abundance and diversity in *Spn*-induced pneumonia mice. Previous study found that gut microbiota can regulate lung immunity to prevent pneumonia, plays an important role in the “gut-lung axis” [15–17]. These results suggested that the underlying mechanism by which SLBZS plays the function to attenuate lung injury in mice may be that SLBZS can regulate the distribution and abundance of intestinal flora.

However, the study has two limitations. First, it is unclear what role the altered intestinal flora plays; second, the mechanism by which the intestinal flora regulates inflammation is unclear.

## Conclusion

SLBZS treatment ameliorates inflammation and lung injury in S. pneumoniae-induced pneumonia mice, which may relate with the increased gut microbiota abundance. SLBZS is a potential drug for treating patients with *S. pneumoniae*-induced pneumonia. However, the relevant mechanisms still require further study.

## Data Availability

The datasets used and/or analysed during the current study are available from the corresponding author on reasonable request.

## Abbreviations

*Spn*: *Streptococcus pneumoniae*
SLBZS: Shen-ling-bai-zhu-san
MPO: Myeloperoxidase
IL-1β: interleukin-1β
TCM: Traditional Chinese medicine
CFU: Colony-forming unit
BALF: Broncho-alveolar lavage fluid
OTUs: Operational taxonomic units
IBD: Inflammatory bowel disease

## References

[1] Cilloniz C, Liapikou A, Martin-Loeches I, Garcia-Vidal C, Gabarrus A, Ceccato A, Magdaleno D, Mensa J, Marco F, Torres A (2018). Twenty-year trend in mortality among hospitalized patients with pneumococcal community-acquired pneumonia. PLoS One.

[2] Amin R, Hatakeyama Y, Kitazawa T, Matsumoto K, Fujita S, Seto K, Hasegawa T (2020). Capturing the trends in hospital standardized mortality ratios for pneumonia: a retrospective observational study in Japan (2010 to 2018). Environ Health Prev Med.

[3] Pick H, Daniel P, Rodrigo C, Bewick T, Ashton D, Lawrence H, Baskaran V, Edwards-Pritchard RC, Sheppard C, Eletu SD (2020). Pneumococcal serotype trends, surveillance and risk factors in UK adult pneumonia, 2013-18. Thorax.

[4] Ghia CJ, Dhar R, Koul PA, Rambhad G, Fletcher MA (2019). Streptococcus pneumoniae as a cause of community-acquired pneumonia in Indian adolescents and adults: a systematic review and meta-analysis. Clin Med Insights Circ Respir Pulm Med.

[5] Kim GL, Seon SH, Rhee DK (2017). Pneumonia and Streptococcus pneumoniae vaccine. Arch Pharm Res.

[6] Amalakuhan B, Echevarria KL, Restrepo MI (2017). Managing community acquired pneumonia in the elderly - the next generation of pharmacotherapy on the horizon. Expert Opin Pharmacother.

[7] Deng D, Chen Z, Jia L, Bu J, Ye M, Sun L, Gen Y, Zhang W, Chen G, Fang B (2019). Treatment of hospital-acquired pneumonia with multi-drug resistant organism by Buzhong Yiqi decoction based on Fuzheng Quxie classical prescription: study protocol for a randomized controlled trial. Trials.

[8] Song Y, Yao C, Yao Y, Han H, Zhao X, Yu K, Liu L, Xu Y, Liu Z, Zhou Q (2019). XueBiJing injection versus placebo for critically ill patients with severe community-acquired pneumonia: a randomized controlled trial. Crit Care Med.

[9] Wang H, Li J, Yu X, Li SY (2018). Integrated traditional Chinese and conventional medicine in treatment of severe community-acquired pneumonia: study protocol for a randomized placebo-controlled trial. Trials.

[10] Li DM, Qi RH, Zhang HC, Liao X, Xie YM, Zhang JH, Zhang BL (2018). Clinical application evaluation and revision suggestions of clinical practice guideline on traditional Chinese medicine therapy alone or combined with antibiotics for community acquired pneumonia. Zhongguo Zhong Yao Za Zhi.

[11] Huang YH, Chen ST, Liu FH, Hsieh SH, Lin CH, Liou MJ, Wang CC, Huang CH, Liu GH, Lin JR (2019). The efficacy and safety of concentrated herbal extract granules, YH1, as an add-on medication in poorly controlled type 2 diabetes: a randomized, double-blind, placebo-controlled pilot trial. PLoS One.

[12] Yang QH, Xu YJ, Liu YZ, Liang YJ, Feng GF, Zhang YP, Xing HJ, Yan HZ, Li YY (2014). Effects of Chaihu-Shugan-san and Shen-Ling-Bai-Zhu-san on p38 MAPK pathway in Kupffer cells of nonalcoholic Steatohepatitis. Evid Based Complement Alternat Med.

[13] Yang L, Song Y, Jin P, Liu Y, Wang Y, Qiao H, Huang Y (2018). Shen-Ling-Bai-Zhu-san for ulcerative colitis: protocol for a systematic review and meta-analysis. Medicine (Baltimore).

[14] Zhang Y, Tang K, Deng Y, Chen R, Liang S, Xie H, He Y, Chen Y, Yang Q (2018). Effects of shenling baizhu powder herbal formula on intestinal microbiota in high-fat diet-induced NAFLD rats. Biomed Pharmacother.

[15] Tamburini S, Clemente JC (2017). Gut microbiota: Neonatal gut microbiota induces lung immunity against pneumonia. Nat Rev Gastroenterol Hepatol.

[16] Peng S, Du TH, Zhang M (2016). Changes in gut microbiota and serum D-lactate level and correlation analysis in children with recurrent pneumonia. Zhongguo Dang Dai Er Ke Za Zhi.

[17] Schuijt TJ, Lankelma JM, Scicluna BP, de Sousa e Melo F, Roelofs JJ, de Boer JD, Hoogendijk AJ, de Beer R, de Vos A, Belzer C (2016). The gut microbiota plays a protective role in the host defence against pneumococcal pneumonia. Gut.

[18] Mahmoodpoor A, Hamishehkar H, Asghari R, Abri R, Shadvar K, Sanaie S (2019). Effect of a probiotic preparation on ventilator-associated pneumonia in critically ill patients admitted to the intensive care unit: a prospective double-blind randomized controlled trial. Nutr Clin Pract.

[19] Ige OM, Okesola AO (2015). Comparative efficacy and safety of CEFIXIME and ciprofloxacin in the management of adults with community-acquired pneumonia in IBADAN, NIGERIA. Ann Ib Postgrad Med.

[20] Minami M, Konishi T, Takase H, Jiang Z, Arai T, Makino T (2017). Effect of Shin'iseihaito (Xinyiqingfeitang) on acute Streptococcus pneumoniae murine sinusitis via macrophage activation. Evid Based Complement Alternat Med.

[21] Cui P, Xin H, Yao Y, Xiao S, Zhu F, Gong Z, Tang Z, Zhan Q, Qin W, Lai Y (2018). Human amnion-derived mesenchymal stem cells alleviate lung injury induced by white smoke inhalation in rats. Stem Cell Res Ther.

[22] Yang Zhaohui, Zou Xiaoguang, Feng Peiqing, Zhan Huaibing, Xiong Dani, Lang Jianmin (2019). Inhibition of the PI3K/AKT Signaling Pathway or Overexpression of Beclin1 Blocks Reinfection of Streptococcus pneumoniae After Infection of Influenza A Virus in Severe Community-Acquired Pneumonia. Inflammation.

[23] Yang X, Liu L, Chen J, Xiao A. Response of intestinal bacterial Flora to the long-term feeding of Aflatoxin B1 (AFB1) in mice. Toxins (Basel). 2017;9. 10.3390/toxins9100317 PMC5666364 29023377

[24] Gu S, Chen D, Zhang JN, Lv X, Wang K, Duan LP, Nie Y, Wu XL (2013). Bacterial community mapping of the mouse gastrointestinal tract. PLoS One.

[25] Ji HJ, Kang N, Chen T, Lv L, Ma XX, Wang FY, Tang XD (2019). Shen-ling-bai-zhu-san, a spleen-tonifying Chinese herbal formula, alleviates lactose-induced chronic diarrhea in rats. J Ethnopharmacol.

[26] Lv WJ, Liu C, Li YF, Chen WQ, Li ZQ, Li Y, Xiong Y, Chao LM, Xu XL, Guo SN (2019). Systems pharmacology and microbiome dissection of Shen Ling Bai Zhu san reveal multiscale treatment strategy for IBD. Oxidative Med Cell Longev.

[27] Zhou Y, Gao H, Mihindukulasuriya KA, La Rosa PS, Wylie KM, Vishnivetskaya T, Podar M, Warner B, Tarr PI, Nelson DE (2013). Biogeography of the ecosystems of the healthy human body. Genome Biol.

[28] Dai W, Wang H, Zhou Q, Feng X, Lu Z, Li D, Yang Z, Liu Y, Li Y, Xie G (2018). The concordance between upper and lower respiratory microbiota in children with mycoplasma pneumoniae pneumonia. Emerg Microbes Infect.

[29] Shankar J, Nguyen MH, Crespo MM, Kwak EJ, Lucas SK, McHugh KJ, Mounaud S, Alcorn JF, Pilewski JM, Shigemura N (2016). Looking beyond respiratory cultures: microbiome-cytokine signatures of bacterial pneumonia and Tracheobronchitis in lung transplant recipients. Am J Transplant.

[30] Wang H, Dai W, Qiu C, Li S, Wang W, Xu J, Li Z, Wang H, Li Y, Yang Z (2016). Mycoplasma pneumoniae and Streptococcus pneumoniae caused different microbial structure and correlation network in lung microbiota. J Thorac Dis.

[31] Prazak J, Iten M, Cameron DR, Save J, Grandgirard D, Resch G, Goepfert C, Leib SL, Takala J, Jakob SM (2019). Bacteriophages improve outcomes in experimental Staphylococcus aureus ventilator-associated pneumonia. Am J Respir Crit Care Med.

[32] Loughran AJ, Orihuela CJ, Tuomanen EI. Streptococcus pneumoniae: invasion and inflammation. Microbiol Spectr. 2019;7:10. 10.1128/microbiolspec.gpp3-0004-2018 PMC6422050 30873934

[33] Smith MW, Schmidt JE, Rehg JE, Orihuela CJ, McCullers JA (2007). Induction of pro- and anti-inflammatory molecules in a mouse model of pneumococcal pneumonia after influenza. Comp Med.

[34] Duell BL, Tan CK, Carey AJ, Wu F, Cripps AW, Ulett GC (2012). Recent insights into microbial triggers of interleukin-10 production in the host and the impact on infectious disease pathogenesis. FEMS Immunol Med Microbiol.

[35] Penaloza HF, Nieto PA, Munoz-Durango N, Salazar-Echegarai FJ, Torres J, Parga MJ, Alvarez-Lobos M, Riedel CA, Kalergis AM, Bueno SM (2015). Interleukin-10 plays a key role in the modulation of neutrophils recruitment and lung inflammation during infection by Streptococcus pneumoniae. Immunology.

[36] Yang Q, Xu Y, Feng G, Hu C, Zhang Y, Cheng S, Wang Y, Gong X (2014). p38 MAPK signal pathway involved in anti-inflammatory effect of Chaihu-Shugan-san and Shen-ling-bai-zhu-san on hepatocyte in non-alcoholic steatohepatitis rats. Afr J Tradit Complement Altern Med.

[37] Kanmani P, Clua P, Vizoso-Pinto MG, Rodriguez C, Alvarez S, Melnikov V, Takahashi H, Kitazawa H, Villena J (2017). Respiratory commensal Bacteria Corynebacterium pseudodiphtheriticum improves resistance of infant mice to respiratory syncytial virus and Streptococcus pneumoniae Superinfection. Front Microbiol.

[38] Dubourg G, Lagier JC, Armougom F, Robert C, Hamad I, Brouqui P, Raoult D (2013). The gut microbiota of a patient with resistant tuberculosis is more comprehensively studied by culturomics than by metagenomics. Eur J Clin Microbiol Infect Dis.

[39] Gauguet S, D'Ortona S, Ahnger-Pier K, Duan B, Surana NK, Lu R, Cywes-Bentley C, Gadjeva M, Shan Q, Priebe GP, Pier GB (2015). Intestinal microbiota of mice influences resistance to Staphylococcus aureus pneumonia. Infect Immun.

[40] Felix KM, Jaimez IA, Nguyen TV, Ma H, Raslan WA, Klinger CN, Doyle KP, Wu HJ (2018). Gut microbiota contributes to resistance against pneumococcal pneumonia in Immunodeficient rag(−/−) mice. Front Cell Infect Microbiol.

[41] Shi Y, Zhai Q, Li D, Mao B, Liu X, Zhao J, Zhang H, Chen W (2017). Restoration of cefixime-induced gut microbiota changes by Lactobacillus cocktails and fructooligosaccharides in a mouse model. Microbiol Res.

[42] Lv W, Liu C, Ye C, Sun J, Tan X, Zhang C, Qu Q, Shi D, Guo S (2017). Structural modulation of gut microbiota during alleviation of antibiotic-associated diarrhea with herbal formula. Int J Biol Macromol.

